# Risk factors of pneumonia in persons with and without Alzheimer’s disease: a matched cohort study

**DOI:** 10.1186/s12877-023-03940-z

**Published:** 2023-04-10

**Authors:** Heli Järvinen, Anna-Maija Tolppanen, Sirpa Hartikainen

**Affiliations:** 1 School of Pharmacy, University of Eastern Finland, Kuopio, Finland; 2 Kuopio Research Centre of Geriatric Care, University of Eastern Finland, PO Box 1627, 70211 Kuopio, Finland

**Keywords:** Alzheimer’s disease, Dementia, Epidemiology, Hospitalization, Pneumonia, Risk factors

## Abstract

**Background:**

Pneumonia is a very common infection in the cognitively impaired adult population, often leading to long-term deterioration, in physical and cognitive performance. Evidence is lacking on whether chronic comorbidities and drug use are risk factors for pneumonia in persons with Alzheimer’s disease (AD). The objective of this study was to investigate the risk factors of pneumonia in community dwellers with and without AD.

**Methods:**

We performed a retrospective register-based study utilizing the Medication Use and Alzheimer’s disease (MEDALZ) cohort, which is based on Finnish nationwide healthcare registers and includes all community dwellers who received a verified clinical diagnosis of AD between 2005 to 2011. This study comprised 69,350 persons with AD and 69,350 persons without AD matched by age, gender, and region of residence. Association between comorbidities, drug use, and hospitalization due to pneumonia were assessed using Cox Regression.

**Results:**

During the follow-up, 25.0% (*n* = 17,105) of the AD cohort and 15.8% (*n* = 10,966) of the non-AD cohort were hospitalized due to pneumonia. Persons with AD had a higher risk of pneumonia also after adjusting for comorbidities (HR 1.76, 95% CI 1.71–1.80). Previous pneumonia was the strongest risk factor for pneumonia in both cohorts. All comorbidities and drug use excluding biological product use were associated with a higher risk of pneumonia, but stronger associations were observed in the non-AD cohort. The risk of hospitalization following psychotropic drug use was proportional to the number of psychotropics utilized.

**Conclusions:**

Pneumonia is a serious, potentially life-threatening illness, and risk factors for pneumonia include several potentially avoidable drugs. In addition, good care of existing comorbidities might prevent pneumonia and related hospitalization.

**Supplementary Information:**

The online version contains supplementary material available at 10.1186/s12877-023-03940-z.

## Background

Pneumonia-related hospitalizations have been increasing during the last decades, especially in older adults [1–3]. This is explained, at least partly, by comorbidities and recurrent cases of pneumonia [1, 2]. Several comorbid conditions are known to predispose to pneumonia [4, 5] as well as the use of, for example, immunosuppressants [4, 6], antipsychotics [7], and benzodiazepines [8]. The relationship between proton pump inhibitors and pneumonia has been debated [9, 10].

The incidence of community-acquired pneumonia (CAP) requiring hospitalization ranges from 267 per 100 000 in the adult population to 1012 per 100,000 for persons over 65 years old [11]. In European studies, the estimated annual cost of pneumonia-related hospitalization in all age groups ranges from €2707 ($3068) to €4000 ($4533) per person [12–14]. According to previous studies, delirium at admission is more common in older adults who are admitted for pneumonia than for matched controls who are admitted for other causes [15].

People with cognitive disorders have a higher hospital admission rate for bacterial pneumonia (34 per 1000 person-years) than those without cognitive disorders (10 per 1000 person-years) [16]. Among persons with cognitive disorders, infections more often result in delirium [17] and may cause further cognitive and functional decline [18, 19]. Persons with cognitive disorders have been found to have more comorbidities than their peers, the most common comorbidities being hypertension, coronary artery disease, stroke, diabetes, and asthma [20–22]. Moreover, cognitive disorder is a risk factor for aspiration pneumonia [23] Previous studies have shown that persons with cognitive disorders have higher mortality within 30 days after pneumonia than those without cognitive disorders [24].

Evidence is lacking on whether comorbidities and utilization of psychotropics, proton pump inhibitors, oral glucocorticoids, or biological products, are risk factors for pneumonia in persons with and without Alzheimer’s disease. To our best knowledge, there are no previous studies on risk factors of pneumonia in community dwellers with Alzheimer’s disease (AD). Thus, we studied the pneumonia risk factors in community dwellers with and without Alzheimer’s disease.

## Methods

### Study population

The MEDALZ (Medication use and Alzheimer’s disease) is a study based on national healthcare registers. It includes 70,718 all community dwellers who have received a clinical diagnosis of AD from 2005 to 2011 in Finland [25]. To identify the persons with AD, we utilized a nationwide Special Reimbursement Register, maintained by the Social Insurance Institution of Finland (SII). This register contains information on entitlement to special reimbursements of drugs for chronic illnesses. SII centrally reviews and confirms the AD diagnosis, which was based on the NINCDS-ADRDA [26] and DMS-IV [27] criteria and includes a computed tomography or magnetic resonance imaging scan and confirmation of the diagnosis by a neurologist or a geriatrician [28]. In this study, each person with AD was matched with a comparison person without AD according to age (± one year), gender, and region of residence at the date of AD diagnosis. The matching was done with incidence density sampling without replacement. This date was assigned as an index date for the persons with AD and the comparison persons.

### Pneumonia

We obtained data on pneumonia diagnoses from Care Register for Health Care, which contains information on inpatient days, including the date and reason for the hospital stay. These diagnoses were based on the International Statistical Classification of Diseases and Related Health Problems 10^th^ revision (ICD-10). Pneumonia diagnoses utilized in this study were the following: J10-12 (viral pneumonia), J13-15 (bacterial pneumonia), J16 (pneumonia due to other infectious organisms, not elsewhere classified), J17 (pneumonia in diseases classified elsewhere), and J18 (pneumonia, organism unspecified). The same codes were used to retrieve information on pneumonia from the Cause of Death register. Previous hospital-treated pneumonia was defined as any of the previous diagnoses six months before AD diagnosis or index date.

### Participant characteristics

Socioeconomic position was defined as the highest occupational social class between 1972 and the index date and the information were obtained from Statistics Finland, as described previously [29]. We retrieved data on comorbidities from Special Reimbursement Register and Care Register for Health Care from 1972 until the index date or AD diagnosis. Relevant comorbidities were defined according to existing literature: chronic obstructive pulmonary disease (COPD), asthma, diabetes, cardiovascular disease, stroke, and chronic renal or liver disease [4, 5]. Special Reimbursement Register included data on the diagnosis of asthma/COPD, diabetes, rheumatoid arthritis or other connective tissue diseases, and cardiovascular disease. More detailed information on definitions of these characteristics is given in Supplementary Table 1.

In addition, data on the use of benzodiazepines and related drugs, antidepressants, antipsychotics, antiepileptics, opioids, biological products, oral glucocorticoids, and proton pump inhibitors were obtained from Prescription Register, which includes information on all purchased and reimbursed prescription drugs from Finnish pharmacies. Drugs were classified according to the WHO Anatomical Therapeutic Chemical (ATC) classification system [30] Drug use was investigated one year before AD diagnosis or index date. Psychotropics include antipsychotics, benzodiazepines, or related drugs and antidepressants. The total number of psychotropics in use for one person could reach a maximum number of three and the number could include drugs from all psychotropic classes. Similarly, antiepileptic and opioid use during the year preceding AD diagnosis or index date was investigated and the utilized ATC codes are listed in Supplementary Table 1. Care Register for Health Care included data on previous hospitalizations due to pneumonia, stroke, and liver or kidney disease. More detailed information on definitions of these characteristics is given in Supplementary Table 1.

**Fig. 1:**
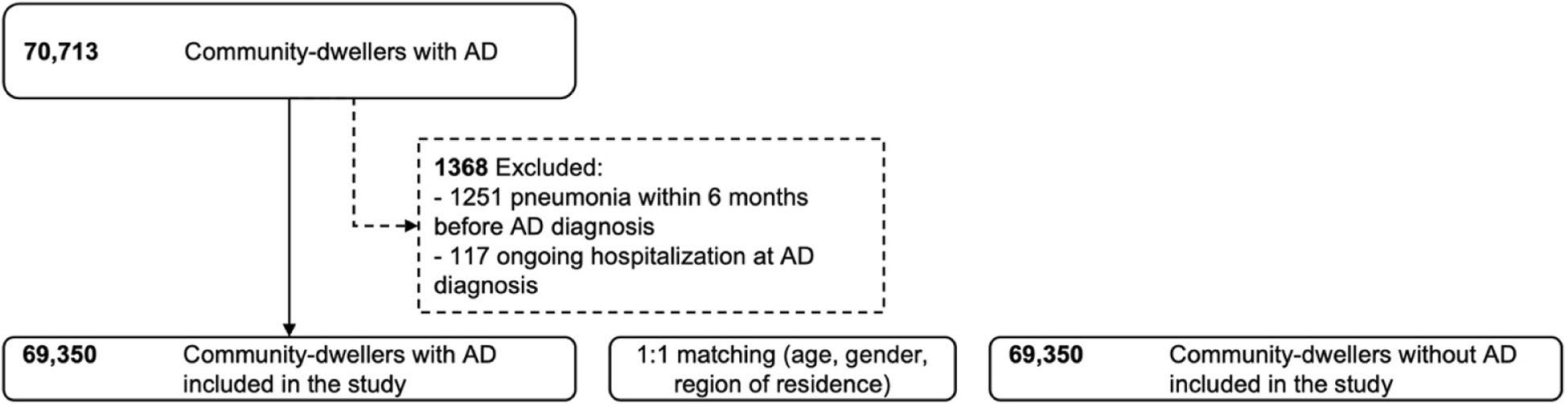
Derivation of the study population. The same exclusion criteria were applied for people with and without AD. For those without AD, the index date for assessing the exclusion criteria was defined based on the matching date

Persons who were hospitalized for pneumonia within a six-month time window before the index date, including those with ongoing hospitalization with pneumonia diagnosis on the index date, were excluded. In addition, persons who received active cancer treatment within six months before the index date were excluded from the data. The study population consisted of 69,350 persons with AD and 69,350 persons without AD. The same exclusion criteria were used for both cohorts. Data sources and periods for identifying exclusion criteria are presented in Supplementary Table 1 and Fig. 1. The follow-up began on the index date and ended on the date of pneumonia diagnosis, death (due to other causes than pneumonia), the beginning of acute cancer treatment, or the end of the data linkage (31.12.2015), whichever occurred first. In addition, the comparison persons were censored on the date of AD diagnosis if that occurred before the other censoring criteria.

### Statistical analyses

χ^2^ test, independent samples T-test, and Mann–Whitney test were utilized to report the *p*-values for categorical and continuous variables. Cox regression was utilized to define the incidence of pneumonia and reported as hazard ratios (HRs) with 95% confidence intervals (CIs). The proportionality assumption was confirmed with Kaplan–Meier curves. Multivariable models were adjusted for: AD, age, gender, socioeconomic position, asthma/COPD, rheumatoid arthritis, cardiovascular disease, diabetes, stroke, liver or kidney disease, benzodiazepine or related drug use, antidepressant use, antipsychotic use, antiepileptic use, opioid use, use of biological products, oral glucocorticoid use, and proton pump inhibitor use. The association of AD and other risk factors with pneumonia were evaluated in the entire study population including both persons with and without AD: To assess whether the association of risk factor was similar in persons with and without AD, models with AD*risk factor interaction term were fitted. As there was statistical evidence for different association between the cohorts, the analyses were also stratified by AD. Statistical analysis was performed using IBM SPSS Statistics for Mac (version 25, IBM Corporation, Armonk, New York, USA). In addition, competing risks regression models with death for other causes than pneumonia as competing risk, according to the method of Fine and Gray were fitted with Stata MP17.0 [31].

### Ethics

The MEDALZ study plan was approved by the register maintainers. All data were pseudonymized before submission to the research team and study participants were not contacted, thus, according to Finnish law, ethics committee approval or informed consent was not required.

## Results

### Characteristics

The study population is described in detail in Table 1. Altogether 8.6% (*n* = 5983) of the AD cohort and 7.4% (*n* = 5126) of the non-AD cohort had had previous hospital-treated pneumonia before the follow-up. Cardiovascular disease, diabetes, and stroke, as well as the use of psychotropics and antiepileptics during one year before AD diagnosis, were more common in the AD cohort. The median follow-up was 4.5 years (IQR 2.5–9.2, range 1 day – 11 years) in the AD cohort and 5.2 years (IQR 3.2–7.2, range 1 day-11 years) years in the non-AD cohort. During the follow-up, 25.0% (*n* = 17,105) of the AD cohort and 15.8% (*n* = 10,966) of the non-AD cohort were hospitalized due to incident pneumonia and the median of hospital days due to pneumonia was 11 days for both groups. The incidence of pneumonia was higher, 5.5/100 person-years in persons with AD and 3.0/100 person-years in persons without AD. Persons with AD were more likely to be censored due to death than those without AD (42.7% and 26.5%, respectively). A detailed description of censoring reasons in both cohorts is provided in Supplementary Table 2.

**Table 1:** Description of the study sample

Variable	AD cohort *n* = 69,350	Non-AD cohort *n* = 69,350	P
Age, mean,years (95% CI)	80.08 (80.03–80.13)	80.04 (79.99–80.09)	0.289
**Gender, n (%)**			matched
Men	24,214 (34.9)	24,214 (34.9)	
Women	45,136 (65.1)	45,136 (65.1)	
**Pneumonia**			
Previous hospital-treated pneumonia, n (%)	5983 (8.6)	5126 (7.4)	< 0.0001
Incident pneumonia	17,105 (25.0)	10,966 (15.8)	< 0.0001
Time from AD diagnosis/index date until first pneumonia (median, d, IQR)	1023 (491–1635)	1091 (499–1810)	< 0.0001
Hospital days due to pneumonia (median, d, IQR)	11 (6–25)	11 (6–23)	0.018
Number of hospital stays due to pneumonia (median, IQR)	1 (1–2)	1 (1–2)	0.685
**Socioeconomic position, n (%)**			< 0.0001
Managerial/professional	14,402 (20.8)	14,676 (21.2)	
Office	5840 (8.4)	5756 (8.3)	
Farming/forestry	13,191 (19.0)	13,810 (19.9)	
Sales/industrial/cleaning	29,558 (42.6)	27,068 (39.0)	
Other	6359 (9.2)	8040 (11.6)	
**Comorbidities, n (%)**			
Asthma/COPD	6042 (8.7)	6332 (9.1)	0.006
Rheumatoid arthritis	2085 (3.0)	2031 (2.9)	0.393
Cardiovascular disease	35,428 (50.8)	34,333 (49.5)	< 0.0001
Diabetes	9205 (13.3)	8003 (11.5)	< 0.0001
Stroke	6706 (9.7)	5810 (8.4)	< 0.0001
Liver or kidney disease	1578 (2.3)	1483 (2.1)	0.083
**Drug use, n (%) CNS-drugs**			
Benzodiazepines and related drugs	18,927 (27.3)	18,265 (26.3)	< 0.0001
Antidepressant	16,129 (23.3)	7269 (10.5)	< 0.0001
Antipsychotic	7315 (10.6)	2390 (3.5)	< 0.0001
Antiepileptic	3859 (5.6)	3192 (4.6)	< 0.0001
Opioid	5515 (8.0)	6035 (8.7)	< 0.0001
**Others**			
Biological products	1051 (1.5)	1267 (1.8)	< 0.0001
Oral glucocorticoid	111 (0.2)	92 (0.1)	0.18
Proton pump inhibitor	13,683 (19.7)	14,003 (20.2)	0.032

*AD* Alzheimer’s disease, *IQR* Interquartile range, *COPD* Chronic obstructive pulmonary disease, *d* Days, *CI* Confidence interval

In both AD and non-AD cohorts, chronic comorbidities (excluding liver and kidney disease in the AD cohort) were more common in persons with incident pneumonia (Table 2). In addition, the use of benzodiazepines and related drugs and antipsychotics, as well as antiepileptics, opioids, oral glucocorticoids, and proton pump inhibitors, was more common in this group.

**Table 2:** Characteristics of persons with and without pneumonia in AD and non-AD cohorts

Variable	AD cohort *n* = 69,350			Non-AD cohort *n* = 69,350		
	Incident pneumonia *n* = 17,105	No incident pneumonia *n* = 52,245	P	Incident pneumonia *n* = 10,967	No incident pneumonia *n* = 58,383	P
Age, mean (years)	80.6	79.9	< 0.0001	82.4	79.6	< 0.0001
**Gender, n (%)**			< 0.0001			< 0.0001
Men	8290 (48.5)	15,924 (30.5)		4763 (43.4)	19,451 (33.3)	
Women	8815 (51.5)	36,321 (69.5)		6204 (56.6)	38,932 (66.7)	
**Socioeconomic position, n (%)**			< 0.0001			< 0.0001
Managerial/professional	3247 (19.0)	11,155 (21.4)		1801 (16.4)	12,875 (22.1)	
Office	1133 (6.6)	4707 (9.0)		727 (6.6)	5029 (8.6)	
Farming/forestry	3688 (21.6)	9503 (18.2)		2753 (25.1)	11,057 (18.9)	
Sales/industrial/cleaning	7525 (44.0)	22,033 (42.2)		4417 (40.3)	22,651 (38.8)	
Other	1512 (8.8)	4847 (9.3)		1269 (11.6)	6771 (11.6)	
**Comorbidities, n (%)**						
Asthma/COPD	2086 (12.2)	3956 (7.6)	< 0.0001	1664 (15.2)	4668 (8.0)	< 0.0001
Rheumatoid arthritis	599 (3.5)	1486 (2.8)	< 0.0001	450 (4.1)	1581 (2.7)	< 0.0001
Cardiovascular disease	9056 (52.9)	26,192 (50.1)	< 0.0001	6419 (58.5)	27,914 (47.8)	< 0.0001
Diabetes	2482 (14.5)	6723 (12.9)	< 0.0001	1560 (14.2)	6443 (11.0)	< 0.0001
Stroke	1919 (11.2)	4787 (9.2)	< 0.0001	1437 (13.1)	4373 (7.5)	< 0.0001
Liver or kidney disease	416 (2.4)	1162 (2.2)	0.114	334 (3.0)	1149 (2.0)	< 0.0001
**Drug use, n (%) CNS drugs**						
Benzodiazepines and related drugs	4920 (28.8)	14,007 (26.8)	< 0.0001	3631 (33.1)	14,634 (25.1)	< 0.0001
Antidepressants	3948 (23.1)	12,181 (23.3)	0.53	1583 (14.4)	5713 (9.8)	< 0.0001
Antipsychotics	2015 (11.8)	5300 (10.1)	< 0.0001	601 (5.5)	1789 (3.1)	< 0.0001
Antiepileptics	1228 (7.2)	2361 (5.0)	< 0.0001	787 (7.2)	2405 (4.1)	< 0.0001
Opioids	1487 (8.7)	4028 (7.7)	< 0.0001	1183 (10.8)	4852 (8.3)	< 0.0001
**Other drugs, n(%)**						
Biological products	269 (1.6)	782 (1.5)	0.48	230 (2.1)	1037 (1.8)	0.021
Oral glucocorticoids	39 (0.2)	72 (0.1)	0.010	25 (0.2)	67 (0.1)	< 0.003
Proton pump inhibitors	3659 (21.4)	10,024 (19.2)	< 0.0001	2820 (25.7)	11,183 (19.2)	< 0.0001

*AD* Alzheimer’s disease, *COPD* Chronic obstructive pulmonary disease, *CNS* Central nervous system

### Risk factors for hospitalization due to pneumonia

Out of all the investigated risk factors, previous hospital-treated pneumonia had the strongest association with incident pneumonia in the whole population with incident pneumonia (aHR 2.25, 95% CI: 2.18–2.33) (Table 3). Male gender (aHR 2.09, 95% CI 2.04–2.14), AD (aHR 1.76, 95% CI 1.71–1.80), all comorbidities (highest aHR in asthma/COPD 1.59 95% CI 1.54–1.65) and drug use (highest aHR for oral glucocorticoids (1.68 95% CI 1.32–2.15) were associated with incident pneumonia. The association of all risk factors except oral glucocorticoids and biological products was different in AD and the non-AD cohorts (P for interaction < 0.05).

**Table 3:** Pneumonia risk factors in both cohorts

Variable	Unadjusted HR (95% CI), Cox regression	Adjusted HR (95% CI), Cox regression*	Adjusted HR (95%CI) in competing risk model*
AD	1.82 (1.78–1.87)	1.76 (1.71–1.80)	1.54 (1.51–1.58)
Age**	1.06 (1.06–1.06)	1.06 (1.06–1.07)	1.03 (1.03–1.04)
Men**	1.81 (1.77–1.85)	2.09 (2.04–2.14)	1.88 (1.84–1.93)
Socioeconomic position, n (%)**			
Managerial/professional	1.00 (reference)	1.00 (reference)	1.00 (reference)
Office	0.93 (0.88–0.98)	1.08 (1.02–1.13)	1.07 (1.04–1.11)
Farming/forestry	1.51 (1.45–1.56)	1.24 (1.20–1.29)	0.91 (0.86–0.95)
Sales/industrial/cleaning	1.29 (1.25–1.34)	1.19 (1.15–1.23)	0.85 (0.82–0.88)
Other	1.19 (1.14–1.25)	1.06 (1.01–1.18)	0.99 (0.94–1.03)
**Comorbidities**			
Asthma/COPD**	1.81 (1.74–1.87)	1.59 (1.54–1.65)	1.54 (1.48–1.59)
Rheumatoid Arthritis**	1.46 (1.37–1.55)	1.37 (1.29–1.46)	1.30 (1.22–1.39)
Cardiovascular disease**	1.41 (1.37–1.44)	1.15 (1.12–1.18)	1.06 (1.03–1.09)
Diabetes**	1.40 (1.35–1.44)	1.21 (1.17–1.25)	1.09 (1.05–1.13)
Stroke**	1.72 (1.66–1.78)	1.30 (1.26–1.35)	1.19 (1.14–1.23)
Liver or kidney disease**	1.68 (1.57–1.81)	1.21 (1.12–1.30)	0.99 (0.92–1.07)
Previous hospital-treated pneumonia**	3.25 (3.14–3.36)	2.25 (2.18–2.33)	1.92 (1.85–1.99)
**Drug use**			
Benzodiazepine or related drug**	1.32 (1.29–1.36)	1.09 (1.06–1.12)	1.10 (1.07–1.13)
Antidepressant**	1.33 (1.30–1.37)	1.09 (1.06–1.13)	1.08 (1.04–1.11)
Antipsychotic**	1.68 (1.62–1.75)	1.32 (1.27–1.38)	1.18 (1.13–1.23)
Antiepileptic**	1.69 (1.62–1.77)	1.39 (1.32–1.45)	1.36 (1.29–1.42)
Opioid**	1.44 (1.38–1.50)	1.16 (1.11–1.21)	1.08 (1.04–1.13)
Biological products	1.18 (1.08–1.29)	1.10 (1.01–1.21)	1.04 (0.95–1.14)
Oral glucocorticoid	2.10 (1.65–2.69)	1.68 (1.32–2.15)	1.42 (1.09–1.85)
Proton pump inhibitor**	1.37 (1.34–1.41)	1.15 (1.12–1.19)	1.10 (1.07–1.13)

^*^adjusted for age, gender, comorbidities, and drug use

^**^P for AD-risk factor interaction < 0.05

Persons with AD had a higher risk of pneumonia than those without AD also after adjusting for comorbidities. Higher risk of pneumonia in persons with AD was still observed after adjusting for competing risk of death (aHR, 95% CI 1.54, 1.51–1.58). The association of other risk factors was weaker in these competing risk analyses, but except for biological products and liver and kidney disease, they were still associated with a higher risk of pneumonia (Table 3).

When risk factor analyses were performed separately in the AD and non-AD cohorts, previous pneumonia was a strong risk factor for pneumonia in both cohorts (persons with AD aHR 2.00, 95% CI: 1.91–2.09, persons without AD aHR 2.61, 95% CI 2.48–2.75) (Table 4). Except for antidepressants, biological products, benzodiazepines or related drugs, and liver or kidney disease that had no association with pneumonia in the AD cohort, all comorbidities, and drug use were associated with the increased risk of pneumonia in both cohorts. The associations were stronger in the non-AD cohort.

**Table 4:** Pneumonia risk factors separated to AD and non-AD cohorts

Variable	AD cohort unadjusted HR (95% CI)	adjusted HR (95% CI)*	Adjusted HR (95%CI) in competing risk model*	Non-AD cohort unadjusted HR (95% CI)	adjusted HR (95% CI)*	Adjusted HR (95%CI) in competing risk model*
Age	1.04 (1.04–1.04)	1.05 (1.05–1.05)	1.02 (1.02–1.02)	1.09 (1.09–1.09)	1.09 (1.09–1.09)	1.06 (1.06–1.06)
Men	2.10 (2.04–2.16)	2.27 (2.20–2.34)	2.01 (1.94–2.07)	1.52 (1.46–1.58)	1.89 (1.82–1.96)	1.73 (1.66–1.80)
**Comorbidities**						
Asthma/COPD	1.66 (1.59–1.74)	1.50 (1.43–1.57)	1.46 (1.40–1.54)	2.06 (1.96–2.17)	1.76 (1.67–1.86)	1.70 (1.60–1.79)
Rheumatoid Arthritis	1.31 (1.21–1.43)	1.30 (1.20–1.41)	1.25 (1.15–1.37)	1.66 (1.51–1.82)	1.50 (1.36–1.65)	1.42 (1.28–1.56)
Cardiovascular disease	1.24 (1.21–1.28)	1.06 (1.03–1.09)	1.00 (0.97–1.03)	1.67 (1.61–1.74)	1.30 (1.25–1.35)	1.19 (1.15–1.24)
Diabetes	1.27 (1.22–1.33)	1.15 (1.10–1.20)	1.06 (1.01–1.10)	1.52 (1.44–1.60)	1.29 (1.22–1.36)	1.15 (1.09–1.22)
Stroke	1.41 (1.35–1.48)	1.15 (1.10–1.21)	1.07 (1.02–1.13)	2.16 (2.04–2.28)	1.52 (1.43–1.61)	1.34 (1.26–1.42)
Liver or kidney disease	1.41 (1.28–1.56)	1.08 (0.98–1.19)	0.93 (0.84–1.03)	2.09 (1.87–2.33)	1.42 (1.27–1.58)	1.05 (0.93–1.18)
Previous hopital- treated pneumonia	2.64 (2.53–2.76)	2.00 (1.91–2.09)	1.74 (1.66–1.83)	4.18 (3.97–4.39)	2.61 (2.48–2.75)	2.17 (2.06–2.30)
**Drug use**						
Benzodiazepines or related drugs	1.14 (1.10–1.18)	1.03 (0.99–1.07)	1.05 (1.01–1.09)	1.59 (1.53–1.65)	1.14 (1.10–1.20)	1.14 (1.09–1.19)
Antidepressants	0.99 (0.95–1.03)	1.01 (0.97–1.05)	1.01 (0.97–1.05)	1.73 (1.64–1.82)	1.30 (1.22–1.37)	1.20 (1.13–1.28)
Antipsychotics	1.29 (1.23–1.35)	1.28 (1.22–1.34)	1.15 (1.10–1.21)	2.15 (1.98–2.33)	1.60 (1.47–1.74)	1.39 (1.27–1.52)
Antiepileptics	1.48 (1.40–1.57)	1.33 (1.26–1.42)	1.29 (1.21–1.37)	1.93 (1.79–2.07)	1.43 (1.33–1.54)	1.42 (1.31–1.53)
Opioids	1.32 (1.25–1.39)	1.13 (1.07–1.20)	1.08 (1.02–1.14)	1.64 (1.55–1.75)	1.20 (1.13–1.28)	1.08 (1.01–1.15)
Biological products	1.15 (1.02–1.30)	1.07 (0.95–1.21)	1.00 (0.88–1.13)	1.30 (1.14–1.48)	1.14 (1.00–1.30)	1.07 (0.93–1.23)
Oral glucocorticoids	2.07 (1.51–2.83)	1.63 (1.19–2.23)	1.29 (0.93–1.81)	2.16 (1.46–3.20)	1.79 (1.21–2.65)	1.71 (1.14–2.57)
Proton pump inhibitors	1.23 (1.19–1.28)	1.10 (1.06–1.14)	1.06 (1.02–1.10)	1.60 (1.54–1.67)	1.23 (1.18–1.29)	1.16 (1.11–1.21)
Any psychotropics	1.10 (1.07–1.14)		1.08 (1–05-1.12)	1.68 (1.62–1.75)		1.29 (1.23–1.34)
Number of psychotropics						
1	1.09 (1.05–1.12)		1.07 (1.03–1.10)	1.58 (1.52–1.65)		1.24 (1.19–1.30)
2	1.13 (1.08–1.18)		1.11 (1.05–1.16)	2.00 (1.87–2.13)		1.39 (1.30–1.50)
3	1.23 (1.12–1.35)		1.19 (1.08–1.31)	2.43 (2.07–2.84)		1.66 (1.41–1.96)
Antiepileptic or opioid use	1.16 (1.11–1.21)		1.26 (1.11–1.43)	1.42 (1.34–1.50)		1.19 (1.12–1.26)
Antiepileptic and opioid use	1.26 (1.11–1.42)		1.08 (1.05–1.12)	1.85 (1.62–2.12)		1.55 (1.34–1.78)

^*^Adjusted for age, gender, comorbidities, and drug use

The use of any psychotropic before the follow-up was associated with a higher risk of pneumonia in both cohorts, and the risk increase was proportional to the number of different psychotropics. The strongest risk factors for pneumonia in the AD cohort were male gender (aHR 2.27, 95% CI: 2.20–2.34) and oral glucocorticoid use (aHR 1.63, 95% CI: 1.19–2.23). In the non-AD cohort, the strongest associations were observed for use of psychotropics from all three psychotropic classes during the year preceding the index date (unadjusted aHR 2.43, 95% CI 2.07–2.84) and for use of two psychotropic classes during the year preceding the index date (unadjusted aHR 2.00, 95% CI 1.87–2.13).

In the competing risk models, in persons with AD cardiovascular diseases, liver and kidney disease, oral glucocorticoids, and biological products were not associated with pneumonia. In persons without AD, the associations of liver and kidney disease and biological products were no longer associated with increased risk of pneumonia in the competing risk models.

## Discussion

In this study, the AD cohort had a higher incidence of pneumonia than the non-AD cohort. Previously experienced hospital-treated pneumonia had the strongest association with incident pneumonia in both cohorts, and several comorbidities and drug use were also associated with incident pneumonia, especially in the non-AD cohort. The use of more than one kind of psychotropic drug during the preceding year of AD diagnosis or index date was a strong risk factor for pneumonia, especially in the non-AD cohort. Nearly all of the associations remained after accounting for the competing interest of death.

A previous study on the incidence of bacterial pneumonia showed a higher risk of hospitalization for persons with cognitive impairment [16]. Our findings are similar; however, we included a broader range of pneumonia diagnoses including viral pneumonia caused by for example influenza virus and other common respiratory viruses. This implies that the risk is higher in persons with AD not only for bacterial pneumonia but also for viral pneumonia.

Although the same risk factors were associated with a higher risk of pneumonia in both cohorts, nearly all risk factors had stronger associations in the AD cohort. This is consistent with our earlier studies on risk factors of hip fracture [32] and mortality [33] in the same MEDALZ study and implies that AD itself is such a strong risk factor for pneumonia that it overrules the relative impact of other risk factors, although these risk factors are still associated with a higher risk of pneumonia in them. The risk of hospitalization from incident pneumonia was strongly associated with the male gender, especially in the AD cohort. According to a systematic review of risk factors of pneumonia, there is no definitive conclusion on the role of gender as a pneumonia risk factor [34]. However, smoking, and associated COPD are significant risk factors for pneumonia [34] and men are more frequent smokers than women [35], which could explain this finding.

We found a strong association between all comorbidities and incident pneumonia, especially in the non-AD cohort. The observed strong association between previous hospital-treated pneumonia and incident pneumonia in both cohorts is likely explained by persistent risk factors for pneumonia, for example, lifestyle factors including smoking and oral hygiene or immunosuppressive conditions, e.g., inflammatory bowel disease, that were not included in our study.

Interestingly, in our study, the use of biological products was not associated with a higher risk of incident pneumonia, but the use of oral glucocorticoids and existing rheumatoid arthritis were associated with a higher pneumonia risk. Oral glucocorticoid use and immunosuppressive therapy have previously been associated with increased pneumonia risk in the adult population [34]. However, users of biological products might be an exceptional group among older adults, especially individuals with AD. It could be possible that the threshold for prescribing biological products for these groups is very high and individuals more prone to pneumonia could be excluded from the users in this process. Furthermore, older adults who utilize biological products could be more frequently vaccinated with influenza and pneumococcal vaccine [36].

Psychotropic use was associated with a higher risk of pneumonia and the risk increase was proportional to the number of different psychotropics. This finding highlights the importance of avoiding psychotropic polypharmacy, as instructed by treatment guidelines [37]. In addition, clinicians should consider deprescribing psychotropics without current indication or if these are prescribed for neuropsychiatric symptoms or sleep disorders [38, 39]. When this is impossible, switching to a safer alternative or dose reduction should be considered. Out of the individual psychotropics, the strongest associations were observed with antipsychotics, especially in the non-AD cohort. A previous systematic review reported an increased risk for pneumonia following the use of first- and second-generation antipsychotics [40] and according to another review of studies conducted in older adults, similar associations were observed in older adults, also those with dementia or AD [41]. Our findings on the association between antipsychotic use at the beginning of the follow-up are in line with these earlier observations. We have also previously shown that antipsychotic users with AD had a higher risk of pneumonia [42]. However, there are important differences between this and the previous study as that study was restricted to persons who initiated antipsychotic use after AD diagnosis, and exposure was modeled as time-dependent, whereas the current study assessed antipsychotic use during one year before AD diagnosis.

Theoretically, benzodiazepines may cause aspiration possibly leading to pneumonia. We found that benzodiazepines and related drugs increased the risk of pneumonia in the non-AD cohort. Our previous study on persons with AD showed an increased risk of pneumonia in benzodiazepine users [43]. However, it must be noted that in this study, drug exposure was assessed from a different time window. A study on nursing home residents, on the other hand, showed a lower risk for pneumonia in persons using benzodiazepines [44]. However, their use is not recommended in older adults for various reasons, including the risk of falls and negative effects on cognition.

We found that antidepressants increased the risk of pneumonia in the non-AD cohort. Previous studies on the use of antidepressants and the risk of pneumonia have shown conflicting results [41]. Antidepressant use was a risk factor for pneumonia in persons with Parkinson’s Disease [45] but another study on nursing home residents did not link antidepressant use to pneumonia [44]. However, contrary to that study, all our study participants were community-dwelling at the beginning of the follow-up. We found antidepressant use to be a risk factor for pneumonia only in the non-AD cohort, which is likely explained by the fact that AD itself is a strong risk factor for pneumonia.

Persons who had used both antiepileptics and opioids during the year preceding the beginning of the follow-up had a higher risk of pneumonia than those who had used only one of these drugs. Both drugs have been linked to pneumonia in previous studies and one of the potential mechanisms for that could be sedation and aspiration [46, 47].

Previous systematic reviews and meta-analyses have shown an increased risk for pneumonia with proton pump inhibitor use [48], but the most recent systematic review raised the question of heterogeneity between reviewed studies as well as both publication and protopathic bias [49]. According to that review, studies that considered protopathic bias did not show an increased risk for pneumonia. We observed a slight increase in the risk for pneumonia following proton pump inhibitor use but it is noteworthy that this study did not include incident proton pump inhibitor users, and this higher risk could be explained by NSAID and/or oral glucocorticoid use.

### Strengths and limitations

A key strength of our study is the utilization of nationwide register-based data which provides valid information on AD diagnoses [50], prescription drug purchases [51], and diagnosed comorbidities [52]. Registers in this study covered all residents regardless of their region or socioeconomic status, minimizing selection bias. Importantly, however, our data did not contain information on several lifestyle factors, for example, smoking, alcohol consumption, nutritional status, and dental hygiene, and therefore we were not able to study their association with pneumonia, or how much the increased risk of pneumonia in persons with AD is explained by these factors. Another limitation of our study is that we could not access information on the severity of AD and thus we were unable to analyze the incidence rate of pneumonia at different stages of the disease.

## Conclusions

Community-dwellers with AD had a higher incidence of pneumonia than community-dwellers without AD. These findings raise the question of whether comorbidities are optimally treated and why the use of psychotropics and especially psychotropic polypharmacy is so prevalent, especially in persons with AD. In clinical practice, we should assess the state of comorbidities and optimize their care in older adults and especially among vulnerable older adults with AD. Furthermore, we need to review drug use, and carefully consider deprescribing of psychotropics without a current indication.

## Supplementary Information

**Additional file 1:** **Supplementary Table 1.** Definitions of comorbidities and drug use and data sources.

**Additional file 2: Supplementary Table 2.** Reasons for censoring in both cohorts.

## Data Availability

The data that support the findings of this study are available from the corresponding author, but restrictions apply to the availability of these data, so they are not publicly available. Data are available from the authors upon reasonable request and with the permission of the register maintainers.

## Abbreviations

AD: Alzheimer’s disease
aHR: Adjusted hazard ratio
ATC: Anatomical therapeutic chemical
CAP: Community-acquired pneumonia
CI: Confidence interval
COPD: Chronic obstructive pulmonary disease
DSM‐IV: Diagnostic and statistical manual of mental disorders
HR: Hazard ratio
MEDALZ: Medication use and Alzheimer’s disease
NINCDS‐ADRDA: National institute of neurological and communicative disorders and stroke and the Alzheimer’s disease and related disorders association
SII: Social insurance institution
