# Supplementary material for: Risk factors of pneumonia in persons with and without Alzheimer’s disease: a matched cohort study
Source: BMC Geriatr. 2023 Apr 10;23:227. doi: 10.1186/s12877-023-03940-z (PMC10084638; doi:10.1186/s12877-023-03940-z)
Supplement: Supplementary file 1 — Additional file 1: Supplementary Table 1. Definitions of comorbidities and drug use and data sources. [file 12877_2023_3940_MOESM1_ESM.docx]

**Supplementary Table 1.** Definitions of comorbidities and drug use and data sources

| **Drug or disease** | **ATC/ICD** | **Data source** | **Time period** |
| --- | --- | --- | --- |
| Antidepressants | N06A | Prescription Register | one year before the index date/AD diagnosis |
| Antiepileptics | N03A | Prescription Register | one year before the index date/AD diagnosis |
| Antipsychotics | N05A (excluding prochlorperazine and lithium) | Prescription Register | one year before the index date/AD diagnosis |
| Asthma/COPD | Reimbursement code 203: ICD E84.0, J41–J45, P27.1 | Special Reimbursement Register | 1972 - index date or AD diagnosis |
| Benzodiazepines and related drugs | N05BA, N05CD, N05CF | Prescription Register | one year before the index date/AD diagnosis |
| Biological products | A07FA, A07FA01, A07FA02, A09AA02, A16AA07, A16AB02, A16AB03, A16AB04, A16AB05,A16AB07, A16AB08, A16AB09, A16AB10, A16AB12, A16AB13, A16AB14, A16AB17, A16AB18, A16AX08, B01AB01, B01AB02, B01AB04, B01AB05, B01AB10, B01AC13, B01AD02, B01AD04, B01AD07, B01AD11, B01AD12, B01AX01, B01AX07, B01AB09, B02AB01, B02AB02, B02BB01, B02BC30, B02BD01, B02BD02, B02BD03, B02BD04, B02BD06, B02BD07, B02BD08, B02BD10, B02BD13, B02BD14, B02BX04, B02BX06, B03XA01, B03XA02, B03XA03, B05AA01, B05AA02, B06AB01, B06AC01, B06AC04, C10AX13, C10AX14, D03BA03, D11AH05, G03GA01, G03GA02, G03GA04, G03GA05, G03GA06, G03GA07, G03GA08, G03GA09, G03GA10, G03GA30, H01AB01, H01AC01, H01AC03, H01AX01, H04AA01, H05AA02, H05AA03, J06BA01, J06BA02, J06BB01, J06BB04, J06BB16, J06BB21, J07AE01, J07AG01, J07AH08, J07AH09, J07AJ52, J07AL01, J07AL02, J07AL52, J07AM51, J07AN01, J07AP01, J07AP03, J07BA01, J07BA02, J07BB01, J07BB02, J07BB03, J07BC01, J07BC02, J07BC20, J07BD52, J07BD54, J07BF03, J07BG01, J07BH01, J07BH02, J07BK01, J07BK02, J07BK03, J07BL01, J07BM01, J07BM02, J07BM03, "J07BX, J07CA02, J07CA06, J07CA09, J07CA10, L03AB07, L03AB08, L03AB10, L03AB11, L03AB13, L04AA04, L04AA23, L04AA24, L04AA25, L04AA26, L04AA28, L04AA33, L04AA36, L04AB01, L04AB02, L04AB04, L04AB05, L04AB06, L04AC02, L04AC03, L04AC05, L04AC07, L04AC08, L04AC10, L04AC11, L04AC12, L04AC13, L04AC14, L04AC16, L04AC17, M03AX01, M05BC01, M05BX04, M05BX05, M09AX02, N02CX07, R03DX05, R03DX08, R03DX09, R03DX10, R05CB13, R07AA02, S01LA04, S01LA05, S01XA19, S01XA22, S01XA24, V01AA02, V01AA03, V01AA05, V01AA07, V01AA11, V01AA20, V03AB14, V03AB37, V03AF07, V04CF01, V04CL,V09DB01, V09EA03, V09EB01, V09GA04, V09HA03, V10XX02, L03AX13, L04AA10, L04AA18 | Prescription Register | one year before the index date/AD diagnosis |
| Cardiovascular disease | Reimbursement codes 201, 205, 206, 207, 211, 213: ICD E78.00, E78.21, I10-I13, I11.0, I13, I15, I20-I22, I24.0, I25, I27, I27.2, I47–I49, I50, I97.1, P29.0 | Special Reimbursement Register | 1972 - index date or AD diagnosis |
| Diabetes | Reimbursement code 103: ICD E10-E14, E89.1 | Special Reimbursement Register | 1972 - index date or AD diagnosis |
| Liver or kidney disease | K70-77, Z49, N17-19 | Care Register for Health Care | 1972 - index date or AD diagnosis |
| Opioids | N02A | Prescription Register | one year before the index date/AD diagnosis |
| Oral glucocorticoids | H02AB (excluding H02AB02, H02AB04, H02AB06, H02AB07) | Prescription Register | one year before the index date/AD diagnosis |
| Pneumonia | J10.0, J11.0, J12-J18 | Care Register for Health Care | 1972 - index date or AD diagnosis |
|  |  |  |  |
| Proton pump inhibitors | A02BC | Prescription Register | one year before the index date/AD diagnosis |
| Rheumatoid arthritis | Reimbursement code 202: ICD A04.6, A39.8, A50.5, D76, H20.1, H30, I33.0, I40.8, J84, K50.9, K51.9, K73.2, K74.3, K75.4, K83.0, L40.5, M02, M05, M06, M08, M13.9, M30–M35, M45, M46.1, M46.9, M86.3, M86.6, M94.1, N03, N04, Q44.2 | Special Reimbursement Register | 1972 - index date or AD diagnosis |
| Stroke | I60-I64 | Care Register for Health Care | 1972 - index date or AD diagnosis |
