# Supplementary material for: Risk factors of pneumonia in persons with and without Alzheimer’s disease: a matched cohort study
Source: BMC Geriatr. 2023 Apr 10;23:227. doi: 10.1186/s12877-023-03940-z (PMC10084638; doi:10.1186/s12877-023-03940-z)
Supplement: Supplementary file 2 — Additional file 2: Supplementary Table 2. Reasons for censoring in both cohorts. [file 12877_2023_3940_MOESM2_ESM.docx]

**Supplementary table 2.** Reasons for censoring in both cohorts

| **Reason for censoring** | **AD cohort, n (%)** | **Non-AD cohort,n (%)** |
| --- | --- | --- |
| End of data linkage | 21,717 (31.3) | 33,303 (48.0) |
| Incident pneumonia | 17,105 (24.7) | 10,966 (15.8) |
| Death | 30,285 (43.7) | 18,381 (26.5) |
| AD diagnosis | 0 (0.0) | 6,056 (8.7) |
| Acute cancer treatment | 243 (0.4) | 644 (0.9) |
| **Total, %** | 69,350 (100.0) | 69,350 (100.0) |

AD; Alzheimer’s disease
